# Increased Plasma Levels of Heparin-Binding Protein on Admission to Intensive Care Are Associated with Respiratory and Circulatory Failure

**DOI:** 10.1371/journal.pone.0152035

**Published:** 2016-03-23

**Authors:** Jonas Tydén, Heiko Herwald, Folke Sjöberg, Joakim Johansson

**Affiliations:** 1 Department of Anaesthesiology and Intensive Care, Östersund Hospital, Östersund, Sweden; 2 Department of Surgical and Perioperative Sciences, Anaesthesiology and Intensive Care, Umeå University, Umeå, Sweden; 3 Department of Cell and Molecular Biology, Lund University, Lund, Sweden; 4 Department of Clinical and Experimental Medicine, Faculty of Health Sciences, Linköping University, Linköping, Sweden; 5 The Burn Center, Department of Hand, Plastic Surgery and Intensive Care, Linköping County Council, Linköping, Sweden; Azienda Ospedaliero-Universitaria Careggi, ITALY

## Abstract

**Purpose:**

Heparin-binding protein (HBP) is released by granulocytes and has been shown to increase vascular permeability in experimental investigations. Increased vascular permeability in the lungs can lead to fluid accumulation in alveoli and respiratory failure. A generalized increase in vascular permeability leads to loss of circulating blood volume and circulatory failure. We hypothesized that plasma concentrations of HBP on admission to the intensive care unit (ICU) would be associated with decreased oxygenation or circulatory failure.

**Methods:**

This is a prospective, observational study in a mixed 8-bed ICU. We investigated concentrations of HBP in plasma at admission to the ICU from 278 patients. Simplified acute physiology score (SAPS) 3 was recorded on admission. Sequential organ failure assessment (SOFA) scores were recorded daily for three days.

**Results:**

Median SAPS 3 was 58.8 (48–70) and 30-day mortality 64/278 (23%). There was an association between high plasma concentrations of HBP on admission with decreased oxygenation (p<0.001) as well as with circulatory failure (p<0.001), after 48–72 hours in the ICU. There was an association between concentrations of HBP on admission and 30-day mortality (p = 0.002). ROC curves showed areas under the curve of 0,62 for decreased oxygenation, 0,65 for circulatory failure and 0,64 for mortality.

**Conclusions:**

A high concentration of HBP in plasma on admission to the ICU is associated with respiratory and circulatory failure later during the ICU care period. It is also associated with increased 30-day mortality. Despite being an interesting biomarker for the composite ICU population it´s predictive value at the individual patient level is low.

## Introduction

Respiratory failure is a serious complication to many different types of critical illness. Acute respiratory distress syndrome (ARDS) is a subdivision of respiratory failure in which the clinical picture of respiratory failure is typical, but the underlying disease can differ greatly.

The pathophysiology of respiratory failure in critical care is not fully understood. Activated leukocytes seem to play an important part and increased vascular permeability causing fluid to accumulate in alveoli is a hallmark of ARDS [1].

Activated granulocytes have been shown to increase vascular permeability in experimental investigations. The mechanism is not fully understood but heparin binding protein (HBP), released from secretory vesicles of adherent granulocytes, is one important mediator of the increased vascular permeability [2].

Decreased oxygenation, measured as a decreased ratio of partial pressure of O_2_ (PaO_2_) in arterial blood divided by the fraction of inspired O_2_ (FiO_2_) in the breathing gas (P:F ratio), is a common measure of respiratory failure. This ratio also defines the different grades of ARDS [3]. In a recent study, concentrations of HBP in plasma were found to correlate with the development of ARDS in injured patients [4], and in another study they were found to be significantly higher in patients with ARDS than in patients with respiratory failure caused by cardiac decompensation [5].

A localized increase in vascular permeability is part of the normal inflammatory process. A more generalised increase, however, leads to loss of circulating blood volume and possibly circulatory instability. Plasma concentrations of HBP were correlated with the development of circulatory instability in patients with sepsis in one study [6]. Concentrations were also increased in patients with shock in another study, but did not differentiate septic shock from other causes of shock [7].

Earlier studies investigating the relation between plasma concentration of HBP and organ failure comprise specific subgroups of patients. Hence they are not suited to draw conclusions on how HBP is associated with organ failure in a mixed ICU-cohort. Moreover, most studies are not consecutive cohorts with relevant controls but rather compares specific, very sick patients with relatively healthy controls.

Our aim was therefore to investigate whether increased concentrations of HBP in plasma was associated with organ failure comprising decreased oxygenation (decreased P:F ratio), ARDS, or circulatory failure in a large consecutive ICU-cohort with mixed diagnoses and to evaluate its value as biomarker in a mixed ICU cohort.

## Materials and Methods

### Setting

This is a prospective, observational study in the 8-bed mixed ICU of Östersund hospital, a 300-bed hospital in Sweden.

### Ethical approval

The study was approved by the regional ethics review board in Linkoping. Verbal consent was given by the patient or next of kin if the patient was not able. Consent was documented on the patients inclusion sheet. Verbal as opposed to written consent was used since many patients were not able to write due to severity of illness. This procedure was approved by the ethics review board due to the observational character of the study.

### Data collection

All patients admitted to the ICU between 1 February 2012 and 31 January 2013 were screened for inclusion. Inclusion criteria were admission to the ICU and presence of or need for an arterial catheter to be inserted. Patients under the age of 18 years and those transferred from other ICUs were excluded. Final diagnosis was set retrospectively by chart review. P:F ratios and SOFA scoring were recorded prospectively day by day.

### Handling of samples and HBP analysis

Blood samples were collected on admission to the ICU and on the two following days. Samples were drawn from the arterial catheter in ethylenediamine-tetra-acetic acid (EDTA) tubes and spun to plasma, which was stored at -80°C until analysed. Concentrations of HPB in plasma were measured as described earlier [8]. Briefly, plates were coated with a mouse monoclonal antibody directed against HBP. Plates were washed with phosphate-buffered saline (PBS) plus 0.05% Tween and blocked with 2% bovine serum albumin in PBS plus 0.05% Tween. Samples of plasma were then diluted and added to plates in duplicate and incubated for 30 minutes at 37°C. Calibration samples of human HBP (0–600 ng/ml) were added in parallel to the plasma samples. After they had been washed, plates were incubated with a polyclonal rabbit antiserum against human HBP, and bound antibodies were detected by incubation with peroxidase-conjugated antibody against rabbit immunoglobulin G. Plates were developed and the optical density was measured at 420 nm. Intra-, and inter-assay coefficient of variation were both less than 10%.

We also recorded levels of C-reactive protein (CRP) and white blood count (WBC) as analysed by our local hospital laboratory.

### Scoring systems

Simplified acute physiology scores (SAPS) 3 were recorded on admission. Sequential organ failure assessment (SOFA) scores were also recorded in parallel and then day 1 and day 2. The last available registration was used to classify the patients as having or not having organ failure. Hence this measurement corresponds to a value most often 48–72 hours after admission to the ICU, the exceptions being patients that were discharged, alive or dead, before that registration were the last available registration was used. Chest radiographs were taken when clinically indicated at the discretion of the intensive care physician. The presence or absence of ARDS was recorded daily using the Berlin criteria [3]. Severe sepsis was defined as suspected or documented infection, two or more systemic inflammatory response syndrome (SIRS) criteria and organ dysfunction [9].

The main outcome variables were ARDS, respiratory and circulatory failure. The outcome variable circulatory failure was defined as a SOFA circulatory sub score of 4. The outcome variable respiratory failure was defined as a P:F ratio < 27 (The oxygenation cut off between mild and moderate in the Berlin ARDS classification).

### Statistical analysis

Data are presented as mean (95% CI) or median (IQR), as appropriate. To compare the significance of differences between groups we used Student’s *t* test or the Mann–Whitney U test, as appropriate. The significances of differences between categorical variables were evaluated using Fisher’s exact test. As plasma HBP values were not normally distributed they were log-transformed. We used the log-transformed figures for statistical analyses but the actual measured HBP values are also shown in tables for clarity and for comparison with other studies.

Comparison of HBP levels between the different predefined diagnosis-groups (sepsis, trauma, intoxication, cardiac arrest, gastrointestinal bleeding, and other surgical or other medical) was made by one-way ANOVA. A post hoc analysis with corrections for multiple comparisons according to Bonferroni was made to find out if any groups were significantly different from any other group. For this analysis, we used the log-transformed value of admission-HBP but the actual (non-normally distributed) measured values are shown in.

Probabilities of less than 0.05 were accepted as significant. Data were analysed with the help of Statistica 12 (StatSoft™ Inc, Tulsa, OK, USA).

## Results

**Fig 1:**
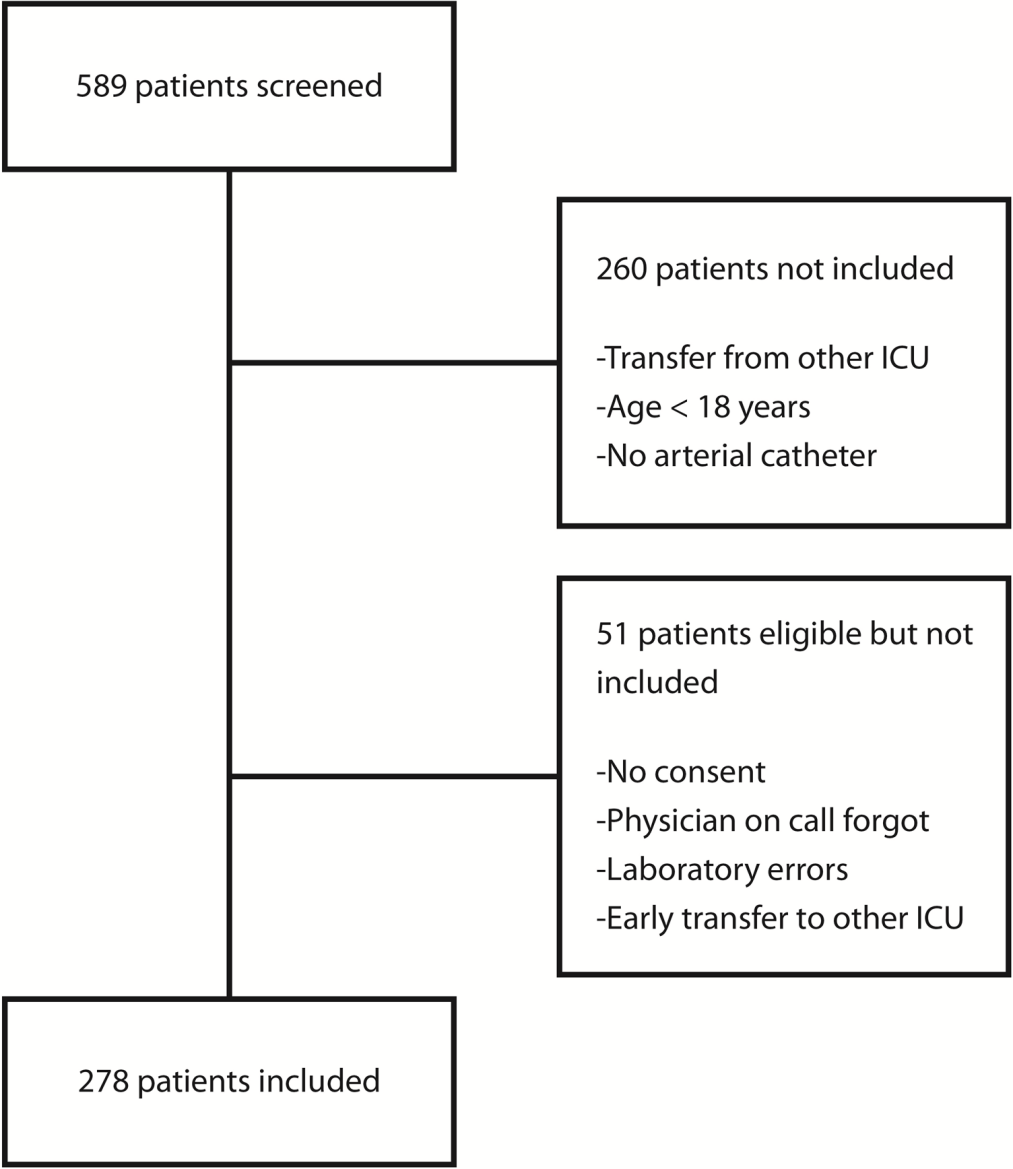
CONSORT table.

Of 589 consecutive patients admitted to the ICU during the study period, 329 were eligible for inclusion and 278 were included (Fig 1). Details of patients, type of admissions, and outcome are shown in Table 1.

**Table 1:** Details of patients.

Age (years)	68 (54–76)
Sex (Male/female)	169/109
log HBP(ng/ml) (mean, 95%CI)	3.79 (3.67–3.90)
HBP (ng/ml)	36.6 (24.5–63.3)
CRP (mg/L) (mean, 95%CI)	83,3 (68,5–98,0)
WBC (x10^9^/L) (mean, 95%CI)	13,7 (12,6–14,7)
Main diagnosis (n, %)	
Sepsis	83 (30%)
Trauma	34 (12%)
Intoxication	11 (4%)
Cardiac arrest	18 (6%)
Gastrointestinal bleeding	11 (4%)
Other surgical	28 (10%)
Other medical	93 (33%)
SAPS 3 score	58 (48–70)
Maximal SOFA score	6 (4–9.5)
ARDS (n, %)	33 (12%)
Duration of stay in ICU (days)	2 (1–3)
ICU mortality (n,%)	33 (12%)
30-day mortality (n, %)	64 (23%)

Data presented as median (interquartile range) where not otherwise indicated or %.

ARDS (acute respiratory distress syndrome)

HBP (Heparin-binding protein)

CRP (C-reactive protein)

WBC (white blood count)

SAPS (simplified acute physiology score)

SOFA (sequential organ failure assessment)

ICU (intensive care unit).

**Fig 2:**
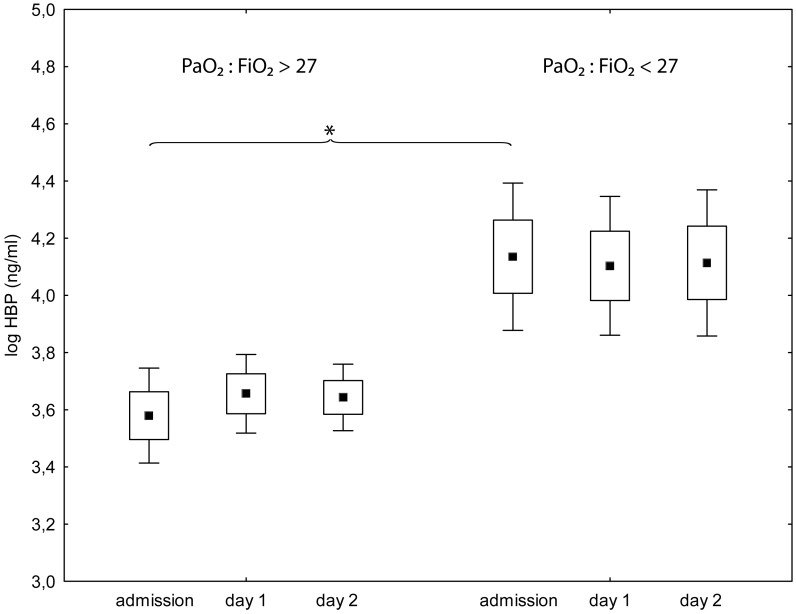
Plasma levels of Heparin binding protein on admission to intensive care and the following two days for patients with an arterial PaO_2_/Fraction of inspired O_2_ of more than 27 kPa (left) or less than 27 kPa (right) on the last registration. Values at admission are significantly higher in patients with PaO_2_/Fraction of inspired O_2_ < 27 (p<0,001). Square, box and bracket indicates mean, standard deviation and 95% confidence interval. * = statistical difference between groups p < 0,05.

**Fig 3:**
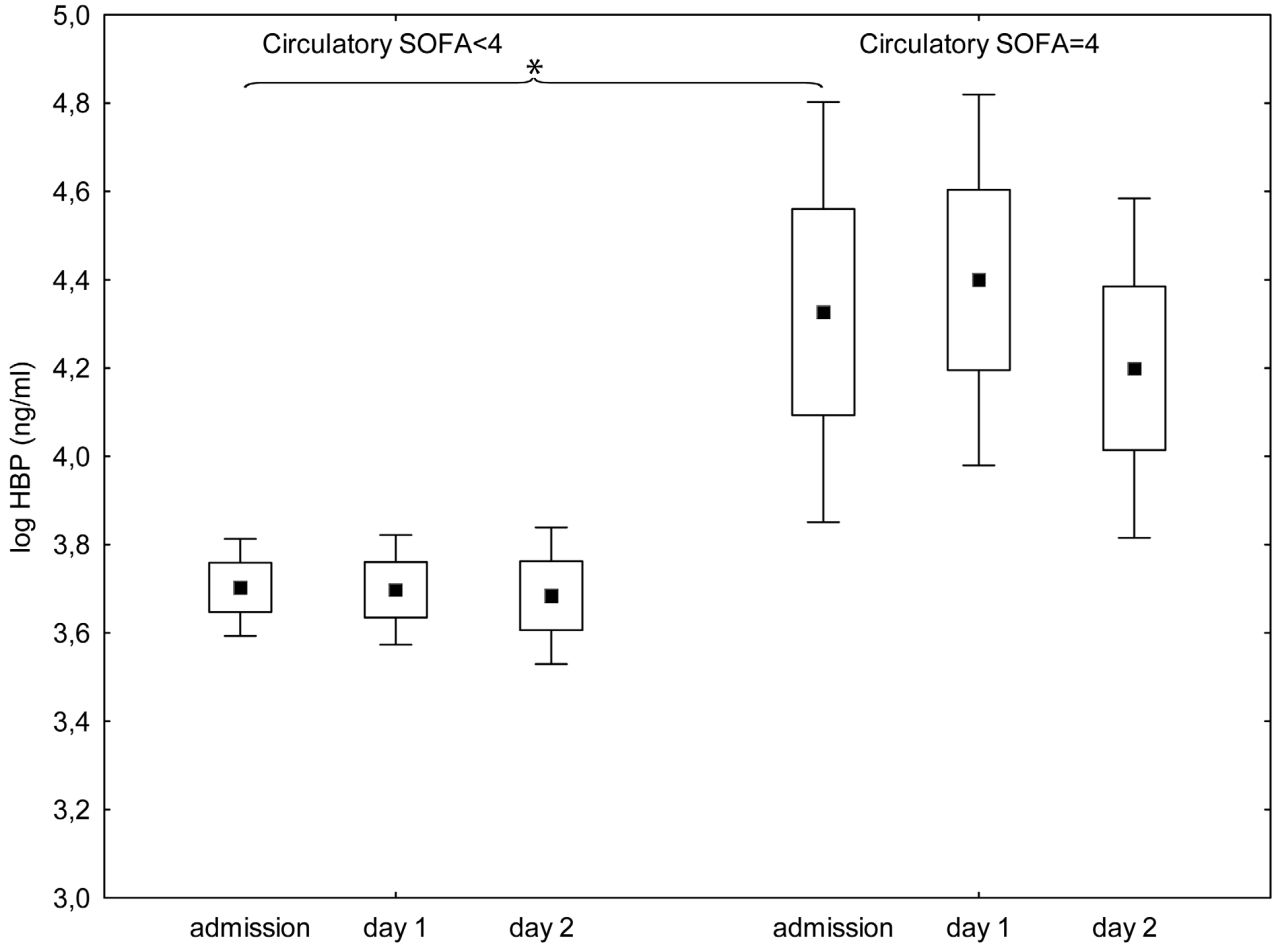
Plasma levels of Heparin binding protein on admission to intensive care and the following two days for patients with circulatory subscore of SOFA less than 4 (left) or 4 (right) on the last registration. Circulatory SOFA indicates circulatory subscore of Sequential organ failure assessment. Values at admission are significantly higher in patients with a circulatory subscore of 4 (p<0,001). Square, box and bracket indicates mean, standard deviation and 95% confidence interval. * = statistical difference between groups p < 0,05.

There was an association between high plasma concentrations of HBP on admission and a P:F-ratio of less than 27 kPa at the last registration(p<0.001) (Fig 2, Table 2). We found no association between plasma concentration of HBP on admission and ARDS (data not shown). There was an association between HBP-concentrations on admission and circulatory SOFA sub score of 4 at the last registration (p<0.001) (Fig 3, Table 2). There was also an association between plasma concentrations of HBP on admission and 30-day mortality (p = 0.002) (Table 3). Using multiple logistic regression for the outcome 30-day mortality, concentrations of HBP on admission did not add to the explanatory value of SAPS 3.

**Table 2:** Patient data in relation to organ failure at last registration (maximum 72 hours after admission).

	Circulatory sub score of SOFA			PaO2:FiO2		
	< 4	= 4	p	> 27 kPa	< 27 kPa	p
Patients, n	234	32		192	79	
Age, years	67(50–76)	73(68–77)	0.010	67(49–76)	70(62–75)	0.072
ICU LOS, days	2(1–3)	4,5(1–9)	<0.001	2(1–3)	3(1–6,5)	0.009
SAPS 3 (mean, CI)	57(55–58)	75(71–79)	<0.001	56(54–58)	65(62–69)	<0.001
Maximal SOFA score	6(4–8)	13(11–16)	<0.001	6(3–8)	9(6–12)	<0.001
HBP, ng/ml	36,4(24–59)	63,5(32–105)	0.007	35,2(23–57)	44,4(30–109)	0.002
log HBP, ng/ml (mean, CI)	3.71(3,60–3,82)	4,33(3,85–4,80)	<0.001	3,64(3,53–3,76)	4.11(3,86–4,37)	<0.001
CRP (mg/L) (mean, CI)	77,6 (61,6–93,5)	93,6 (49,4–137,8)	0.486	68,2 (51,0–85,3)	111 (83,6–140,0)	0.007
WBC (x10^9^/L) (mean, CI)	13,9 (12,8–15,1)	12,4 (8,4–16,4)	0.348	14,9 (12,7–15,2)	13,1 (10,9–15,3)	0.466
ICU mortality (n, %)	17 (7,3%)	15 (46,9%)	<0.001	16 (8,3%)	17 (21,5%)	0.004
30-day mortality (n, %)	42 (17,9%)	19 (59,4%)	<0.001	38 (19,8%)	25 (31,6%)	0.041

Data presented as median (interquartile range) where not otherwise indicated or %.

HBP (Heparin binding protein)

CRP (C-reactive protein)

WBC (white blood count)

SAPS (simplified acute physiology score)

SOFA (sequential organ failure assessment)

ICU (intensive care unit)

LOS (length of stay).

**Table 3:** Patient data in relation to mortality (at 30 days from admission).

	dead	alive	p
Patients, n	64	214	
Age, years	75 (68,5–79)	65 (49–75)	<0.001
SAPS 3 (mean, CI)	73,0 (70,0–76,0)	54,6 (52,8–56,4)	<0.001
Maximal SOFA score	10 (7–13)	6 (3–8)	<0.001
HBP, ng/ml	47,7 (31,2–100,8)	36,4 (23,1–57,1)	<0.001
log HBP, ng/ml (mean, CI)	4,1 (3,86–4,34)	3,7 (3,57–3,81)	0.002
CRP (mg/L)	95,3 (63,3–127,2)	79,7 (62,9–96,4)	0.380
WBC (x10^9^/L)	15,6 (12,7–18,4)	13,1 (12,0–14,2)	0.049

Data presented as median (interquartile range) where not otherwise indicated or %.

HBP (Heparin binding protein)

CRP (C-reactive protein)

WBC (white blood count)

SAPS (Simplified acute physiology score)

SOFA (sequential organ failure assessment)

ICU (intensive care unit)

LOS (length of stay).

Looking only at the patients with severe sepsis, there was an association between concentrations of HBP on admission and circulatory SOFA sub score of 4 at last registration in the 48–72h interval after admission (p = 0.012) but not with P:F-ratio less than 27 kPa (p = 0.2). In non septic patients there was an association between concentrations of HBP on admission and a P:F-ratio less than 27 kPa at last registration in the 48–72h interval after admission (p = 0.003) but not with circulatory SOFA sub score of 4 (p = 0.07).

**Fig 4:**
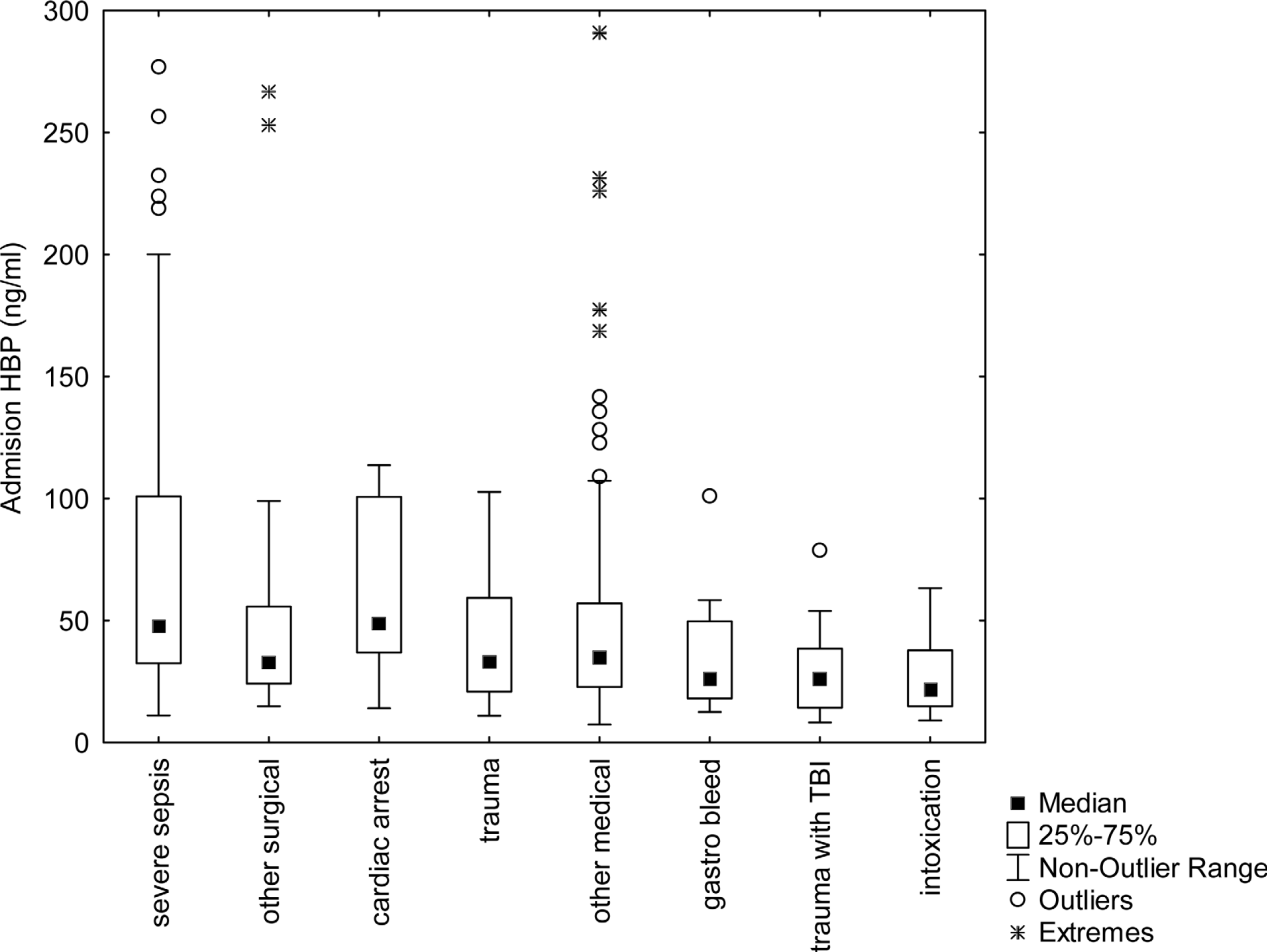
Heparin binding protein (HBP) concentration at admission in the different groups. Log admission-HBP was higher in the group with severe sepsis when compared with the groups ‘other medical’ (p = 0.009) and ‘trauma with TBI’ (p = 0.013). Square, box and brackets indicate median, IQR and non-outlier range. There are three extreme outliers in the severe sepsis group that are not shown (out of range of Fig). (TBI = Traumatic brain injury).

Log admission-HBP was higher in the group with severe sepsis when compared with the groups ‘other medical’ (p = 0.009) and ‘trauma with TBI’ (p = 0.013). The actual measured (non log value) for the different groups are shown in Fig 4.

**Fig 5:**
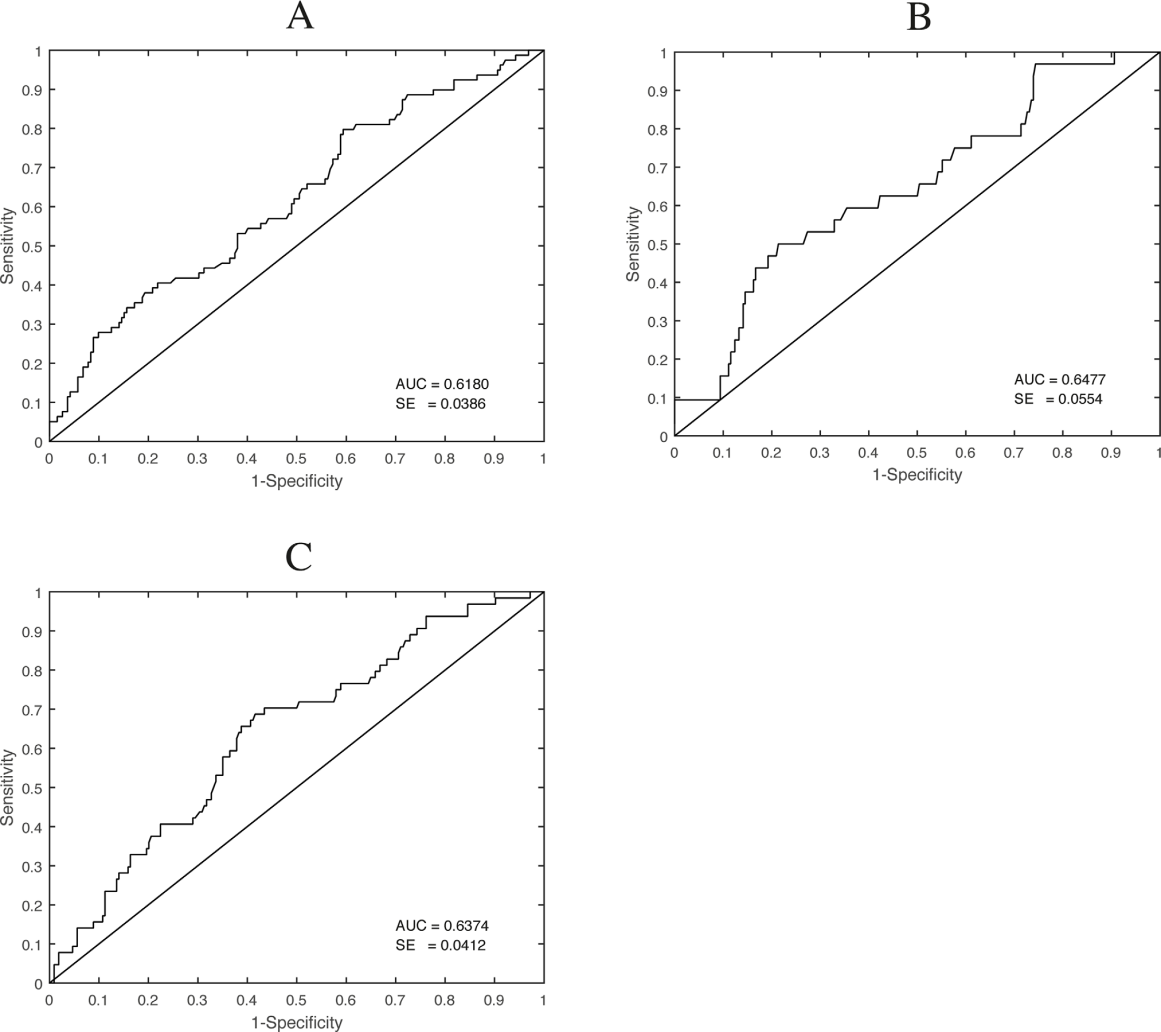
ROC curves for sensitivity and specificity of plasma levels at admission of Heparin binding protein for a P:F ratio of < 27 at last registration (A), for a circulatory subscore of SOFA of 4 at last registration (B) and for 30-day mortality (C).

ROC-curves looking at sensitivity and specificity of plasma levels of HBP at admission showed areas under the curve of 0,61 (0,54–0,69) for a P:F ratio less than 27 kPa at last registration, 0,65 (0,54–0,76) for a circulatory subcore of SOFA of 4 at last registration and 0,64 (0,56–0,72 for 30-day mortality (Fig 5).

## Discussion

Previous experimental research has identified HBP as an important mediator of increased vascular permeability in inflammation [2], and clinical studies have indicated a correlation between plasma concentrations of HBP and shock or ARDS in subsets of patients [4, 5, 7]. We show in this study that plasma concentrations of HBP on admission to the ICU are associated with respiratory failure (p<0,001), circulatory failure (p<0,001) and mortality (p = 0,002) but that the value of HBP as a biomarker in the individual patient is low in an ICU population with a large variety of diagnoses.

Several investigations have pointed to a connection between early organ failure and relevant end points, such as 30-day mortality [10, 11]. HBP sampled on admission to ICU may possibly be associated with organ failure of early onset but probably not later organ failure. There are reasons to think that different pathophysiological mechanisms are responsible for early as opposed to late organ failure [12], and this study relates to early organ failure. We used organ failure at 48 to 72 hours after admission as our primary endpoint, but we also looked at mortality.

In contrast to the previous studies in trauma patients [4] and patients with ARDS [5] we found no correlation between plasma concentrations of HBP and the development of ARDS. When looking at the P:F-ratio, however, there was a significant association between concentrations of HBP and the degree of respiratory failure(p<0,001). Part of this discrepancy is accounted for by the fact that we did not take daily chest radiographs as a routine, which most likely made us miss bilateral opacities. This had an impact on our incidence of definitive ARDS (only 12%) and suggests that patients with ARDS were missed.

In a recent study that compared patients with septic shock and shock of other origins there was no difference between the groups with regard to plasma concentrations of HBP, while concentrations in both groups were significantly higher than in controls [7]. This result suggests that high concentrations may be associated with severity of illness rather than the underlying condition, and this was further supported in a recent study of the subgroup of patients in whom spontaneous circulation returned after cardiac arrest [13]. In our material plasma HBP-levels trended higher in patients with severe sepsis and after cardiac arrest, Fig 3. These groups were, however, also more severely ill as defined by SAPS 3 (data not shown).

The concentration of HBP in the plasma of febrile patients who presented to the emergency department was a good predictor of circulatory instability as a result of sepsis in a study by Linder et al [6]. In line with these data the admission HBP-concentrations in our cohort were associated with high circulatory SOFA sub scores both when looking at all patients (p<0,001) and when looking at the subset with severe sepsis (p = 0,012).

In our series, plasma concentrations of HBP were associated with 30-day mortality (p = 0,002). Some previous studies [5, 6] have showed similar results, while others [7, 14] did not. This is probably the result of differences in the way we selected patients and differences in the timing of sampling.

Our study has several strengths. It includes a wide variety of diagnoses that makes the results more applicable to a mixed ICU. Early inclusion, with initial blood-sampling on admission, makes the results more clinically interesting since this is how real patients typically are sampled. The early inclusion probably also explains why we found higher concentrations of HBP in plasma than earlier studies. Earlier studies have limitations like later sampling, inclusion special subgroups of patients only and comparisons with much healthier patients. We aimed to include all consecutive, eligible patients at our ICU but missed 51/329 due to lack of resources. Since we compare our sickest patients with the relatively healthy ones cared for during the same time, our study resembles how a biomarker would be used clinically.

One limitation of our study is that by including patients on admission to the ICU there is a selection of patients who are severely ill. The results might not be representative of patients who present, for example, to the emergency department. Our cohort is even sicker than our typical mean ICU-patient since we included only patients with an arterial catheter. Some patients are cared for in the ICU without such a catheter but these are most likely without organ failure and low mortality. Another weakness was the problem of defining ARDS without routine chest radiographs that we have discussed above. There were a number of outliers with extremely high HBP in plasma. This is a problem if HBP is to be used in clinical routine.

The association that we, and others, have shown between plasma HBP and different measures of morbidity and mortality is interesting but does not prove a cause and effect relation between HBP and organ failure. Therefore it is interesting to look at a recent experimental study of sepsis induced respiratory failure in pigs where the authors found lower levels of HBP and lower degree of respiratory failure in the group where endothelin-1 was blocked [15]. A study where the action of HBP is blocked in an animal model would thus indeed be interesting.

We conclude that plasma concentration of HBP at admission to intensive care is associated with early development of respiratory and circulatory failure as well as to 30-day mortality. Whether measurement of HBP could help to identify those patients who were in need of early increased attention, monitoring, and circulatory and respiratory support is an interesting field for future research but our results suggest that the specificity and sensitivity for identification of patients at risk is low. Further investigations into a possible causal relation between leukocyte activation, secretion of HBP, and organ failure is also of significant interest.

## Supporting Information

S1 Dataset Registered data for all patients. (XLSX)
